# Odontonutraceuticals: Pleiotropic Phytotherapeutic Agents for Oral Health

**DOI:** 10.3390/ph9010010

**Published:** 2016-02-25

**Authors:** Elena Maria Varoni, Marcello Iriti

**Affiliations:** 1 Dipartimento di Scienze Biomediche, Chirurgiche ed Odontoiatriche, UniversitàdegliStudi di Milano, Milan 20142, Italy; 2 Dipartimento di ScienzeAgrarie e Ambientali, Università degli Studi di Milano, Milan 20133, Italy

**Keywords:** nutrition, food sciences, herbal medicine, drug delivery, phytochemicals, transmucosal absorption, multi-targeted activity

## Abstract

This brief commentary aims to focus on the urgency of further clinical research on phytotherapy in dentistry, and, noteworthy, to propose, for the first time, to the best of our knowledge, the term “odontonutraceuticals” to identify those phytochemicals relevant for the prevention and the treatment of oral diseases. A valuable impact is expected on nutritional, dental and biomedical sciences, suggesting the use of the suffix "odonto-" to define a specific field of nutraceutical research.

As the incidence of nutrition-related diseases is increasing greatly, along with the scientific and media’s interest for dietary styles and herbal medicine, nutritional-based complementary clinical approaches acquire, today, considerable relevance. A current example comes from “Expo Milano 2015”, the non-commercial Universal Exposition, which will be mainly focused on the problems related to nutrition and health, and on the food resources of our planet. To date, abundant preclinical literature and epidemiological data exist in supporting beneficial effects of some plant-derived compounds, namely phytochemicals, towards general well-being, as well as in the prevention and treatment of a number of diseases [1].

Dentistry is not exempt from this topic. Due to the recent interest for a phytotherapeutic approach to dentistry [2,3], we would like, here, to introduce, to the best of our knowledge for the first time, the term “odontonutraceuticals”, in order to identify those phytochemicals relevant for the prevention and the management of oral diseases. “Odonto” arises from the Greek word *odòntos* for “tooth”, while the term *nutraceuticals* refers to bioactive phytochemicals, which can provide health-promoting effects [4].

Since ancient times, plants have been an exemplary source of pharmaceuticals and a plethora of preclinical and epidemiological studies has documented the potential of nutraceuticals in the prevention of the major chronic and degenerative diseases, by virtue of their cardioprotective, neuroprotective and anticancer properties [5]. Similarly, (odonto)nutraceuticals may play a significant role in the care of dental patients, as many oral diseases include complex and multifactorial disorders, often involving multiple deregulated pathways. In these terms, odontonutraceuticals can represent promising and pleiotropic phytotherapeutic agents in dentistry, because of their ability to regulate different molecular and biochemical targets [6]. Interestingly, recent findings suggest that plant extracts, composed by a number of bioactive components, are more effective than individual compounds, because of additive and/or synergistic effects. Relevant examples of odontonutraceuticals include green tea, grape and cocoa seed extracts, rich in polyphenols, particularly catechins, a group of flavonoids, and proanthocyanidins (Figure 1) [7,8]. Nonetheless, *Aloe vera*-based gels showed interesting properties in mucosal wound healing, even with a certain capacity to promote pain control in oral lichen planus patients, as reported by a Cochrane review on this topic [9].

However, despite the promising preclinical evidences and the presence of relevant epidemiological and clinical data, further research on nutraceuticals in dentistry still needs to be carried out, mainly to better elucidate the potential role of confounding factors and compound pharmacokinetics [2]. In particular, the low oral bioavailability of phytochemicals remains, to date, the major drawback of this approach. After oral administration, to achieve the effective concentrations at the target sites, the bioactive components of ingested food have to pass a series of mucosal barriers, at gastro-enteric level, and resist, in active form, to biotransformation by phase I and II enzymes, including the hepatic first-pass metabolism, as well as by bacteria of the intestinal microbial flora. In this view, the efficacy of nutraceuticals can be greatly improved by innovative local drug delivery systems [1], which can enhance their transmucosal absorption. Buccal mucosa represents a recognized and very attractive topical route of administration, for both local action and systemic absorption of (odonto)nutraceuticals. Many pharmaceutical formulations have been recently developed to successfully produce more and more muco-adhesive carriers in order to enhance absorption of phytochemicals, including nanoparticle-enriched films, hydrogels, sprays and patches [1]. These carriers would improve the topical activity of odontonutraceuticals on oral cavity, as well as their systemic effects, due to increased absorption.

To conclude, odontonutraceuticals are expected to represent promising agents towards multi-factorial oral diseases and to acquire more and more importance within the scientific/clinical scenario. Their efficacy, in dental patients, needs to be supported by further evidence and, in these terms, we strongly emphasize a systematic approach to verify phytochemical efficacy, rising the pyramid of evidence-based dentistry, where the preclinical research is the essential prerequisite for clinical studies, which, in turn, represent the apex of evidence [10]. Blind randomized clinical trials, designed to compare odontonutraceuticals with current gold-standard treatments, wherever possible, will greatly improve both scientific knowledge and clinical relevance.

## Figures and Tables

**Figure 1:**
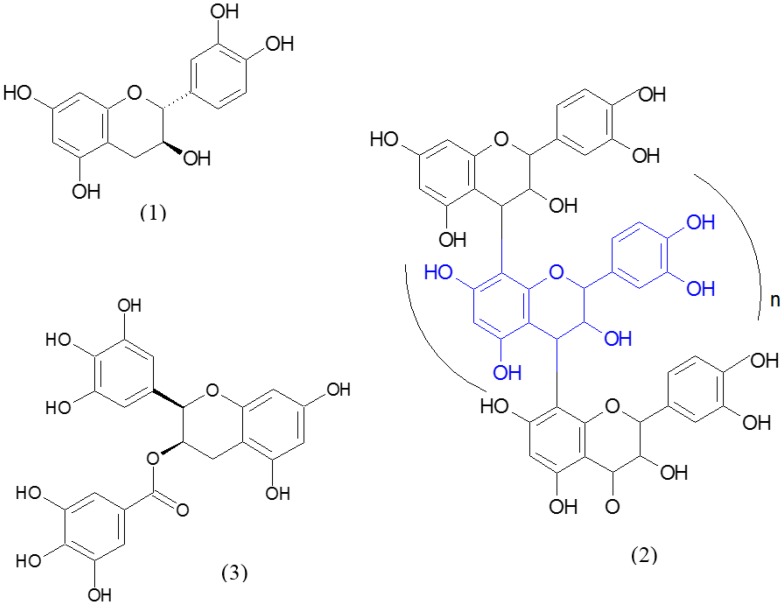
Polyphenols relevant for oral health: (**1**) (+)-catechin and (**2**) epigallocatechin-3-gallate are flavan-3-ols, a group of flavonoids, whereas (**3**) proanthocyanidins arise from the oligomerization or polymerization of flavan-3-ol units.
